# Functional study of the association of *CHI3L1* polymorphisms with asthma susceptibility in the Southwest Chinese Han population

**DOI:** 10.1042/BSR20182008

**Published:** 2019-05-14

**Authors:** Guo Chen, Miao-Miao Zhang, Yu Wang, Shou-Quan Wu, Ming-Gui Wang, Jian-Qing He

**Affiliations:** 1 Department of Respiratory and Critical Care Medicine, West China Hospital, Sichuan University, Chengdu, Sichuan, China; 2 Department of Geriatrics, Sichuan Academy of Medical Sciences & Sichuan Provincial People’s Hospital, Chengdu, Sichuan, China; 3 Chinese Academy of Sciences Sichuan Translational Medicine Research Hospital, Chengdu, Sichuan, China

**Keywords:** asthma, CHI3L1, function, polymorphism, susceptibility

## Abstract

**Background:** Chitinase 3-like 1 (*CHI3L1*) is involved in the Th2 cell mediated pathway, tissue remodeling and fibrosis. Correlations of *CHI3L1* gene polymorphisms with asthma in previous studies have been inconsistent. The present study was designed to investigate the association between *CHI3L1* polymorphisms and asthma in the southwest Chinese Han population. **Methods:** Two single nucleotide polymorphisms (SNPs), rs4950928 and rs10399931, were genotyped in 410 asthma patients and 418 healthy controls from Southwest China. Dual-luciferase reporter gene analysis was performed to detect allele-dependent promoter activity of *CHI3L1* variants in HEK293 cells. Real-time quantitative PCR was applied to detect the relative mRNA expression associated with different genotypes of *CHI3L1* rs10399931. A meta-analysis was performed using data collected from previously published reports and the present study. **Results:** No significant association was found between rs4950928 and asthma. The rs10399931 CT/TT genotype increased the risk of asthma under the dominant model (*P* = 0.031, OR = 1.428, 95% CI, 1.033–1.974), while the CT genotype showed the same tendency under the heterozygous model (*P* = 0.003, OR = 1.680, 95% CI, 1.186–2.380). No statistically significant difference was found between alleles T and C of rs10399931in the dual-luciferase reporter gene analysis (*P* = 0.201). The rs10399931 CT/TT genotypes reduced the relative mRNA expression detected by real-time quantitative PCR (*P* = 0.002). There was no significant association between the *CHI3L1* rs4950928 polymorphism and the risk of asthma in the meta-analysis. **Conclusion:** In the southwest Chinese Han population, the *CHI3L1* rs10399931 CT/TT genotypes may increase the risk of asthma. rs10399931 may be a functional variant of *CHI3L1* due to its effect on mRNA expression.

## Introduction

Asthma is a complex airway disorder characterized by chronic airway inflammation, bronchial hyper-responsiveness and reversible airway obstruction. Currently, 300 million patients worldwide are suffering from this disease and the number may increase to 400 million by 2025 [1].

Although the exact etiology of asthma is still uncertain, it is considered as a heterogeneous disease caused by the interaction of genetic and environmental factors. The incidence of asthma varies in different countries and ethnicities [2]. Chitinase 3-like 1 (C*HI3L1*), localized at chromosome 1q32.1, encodes the YKL-40 protein. *CHI3L1* was identified as an asthma susceptibility locus that was also related to airway hyper-responsiveness and decline in lung function in a population of European descent in a genome-wide association study (GWAS) [3]. *CHI3L1* and YKL-40 protein are involved in the Th2 cell mediated inflammatory pathway, tissue remodeling and fibrosis [4,5]. However, the genetic association study results among different populations were controversial. The objective of this present study was to investigate the association of common variants in *CHI3L1* with adult asthma in a southwest Chinese Han population.

## Materials and methods

### Study population

All subjects were unrelated Chinese Han individuals recruited from the West China Hospital of Sichuan University from 2014 to 2016. Informed consent was obtained from every participant in this case and control study which was approved by the ethical committee of the West China Hospital of Sichuan University (ethics approval number, 2013-23).

The 410 asthmatic patients were diagnosed by physicians in the respiratory clinic according to the criteria of the Global Strategy for Asthma Management and Prevention [2]. Subjects with a respiratory disease other than asthma, a tumor, an immune disease or those using hormones or immunosuppressive drugs were excluded. The 418 controls were healthy and collected from the physical examination center in the same hospital.

### Information on potential confounders

A blood sample was drawn from every subject and stored in a −80°C freezer. Clinical information including sex, age, body mass index (BMI), smoking history and the age of asthma onset, was collected from the medical records, questionnaire and telephone follow-up. Spirometry was performed in the pulmonary function test department of the West China Hospital. The forced expiratory volume in the first second (FEV1) and forced vital capacity (FVC) were measured and expressed as percent of predicted (FEV1PP and FVCPP).

### Gene selection and genotyping

rs4950928 and rs10399931 were selected after literature review. The minor allele frequency (MAF) of the two single nucleotide polymorphisms (SNPs) were 0.146 and 0.359, respectively, with *r*^2^ <0.8 in Han Chinese in Beijing, China (CHB) in 1000 genomes (http://grch37.ensembl.org/Homo_sapiens/Info/Index). Genomic DNA was isolated from the peripheral blood using a genomic DNA purification kit (Axygen Scientific Inc, Union City, CA, U.S.A.). SNPs were genotyped by Genesky Bio-Tech Co., Ltd (http://geneskybiotech.com/index.html) using the SNPscan™ multiplex SNP genotyping technique. The probes and primers were designed by the SpectroDESIGNER software (Sequenom, Sequenom Inc, San Diego, CA, U.S.A.). In addition, 5% of random samples were repeatedly genotyped with a concordance rate of 100%. The JASPAR database (http://jaspar.genereg.net/) and F-SNP database (http://compbio.cs.queensu.ca/F-SNP/) were used to predict the function of asthma susceptibility SNPs.

### Functional analysis of asthma-associated *CHI3L1* polymorphisms

The allele-dependent promoter activity of *CHI3L1* was detected by the dual-luciferase reporter gene system. HEK293 cells were transfected with the Firefly luciferase reporter plasmid pGL3-basic (Promega, USA) under the control of the *CHI3L1* promoter region containing each allele of rs10399931. The pRL-CMV renilla luciferase reporter plasmid (Promega, USA) was cotransfected for normalization of transfection efficiency, and Dual-Luciferase reporter assays were read 24 h later with a GloMax 96 Microplate Luminometer. RNAs of 52 subjects were extracted from the whole blood stored in Tempus™ Blood RNA Tubes by Terpus™ Spin RNA Isolation Reagent Kit (Thermo Fisher Scientific, USA), then reverse transcribed to cDNA by PrimeScript™ RT reagent Kit with gDNA Eraser (Perfect Real Time) (Takara, Japan). Real-time quantitative PCR was applied to detect the relative mRNA expression with different genotypes of *CHI3L1* rs10399931 with QuantiNova™ SYBR Green PCR Kit (QIAGEN, Germany).

### Meta-analysis

We searched the electronic databases of PubMed, Embase, China National Knowledge Infrastructure (CNKI, www.cnki.net) and Wanfang database (www.wanfangdata.com.cn) for all the eligible literature published up to February 2019 using the key search terms: (‘CHI3L1’ or ‘chitinase 3–like 1’), (‘single nucleotide polymorphism’ or ‘SNP’ or ‘polymorphism’ or ‘variation’ or ‘mutation’) and (‘asthma’ or ‘asthmatic’). The language was restricted to English or Chinese but there was no other limitation of any type in the literature searches. In addition, we manually searched the references of relevant publications. All analyses in the current meta-analysis were based on previously published studies and our present study. The inclusion criteria were: (1) studies with a case-control design and the diagnosis criteria of asthma according to international criteria [2]; (2) studies that evaluated the correlation between *CHI3L1* polymorphism and risk of asthma; (3) studies that provided sufficient data on the genotypic and allelic distributions of *CHI3L1*polymorphisms. Exclusion criteria were as follows: (1) studies without control groups; (2) studies with no available data reported and extracted; (3) reviews, letters, abstracts, animal experiments, GWAS studies, epigenetic studies, or overlapping and irrelevant studies. Two independent authors (Guo Chen and Miao-Miao Zhang) extracted the detailed information and data from each primary study. The correspondent author (Jian-Qing He) reviewed these articles, if there was any doubt.

### Statistical analysis

All analyses were performed using the Statistical Package for the Social Sciences (SPSS, SPSS Inc., Chicago, IL, USA), version 17.0. All data were expressed as the mean ± standard deviation. Comparisons of the cases and controls were conducted using Pearson’s Chi-squared test. Continuous variables were analyzed using the Mann–Whitney *U* test. Genotype distributions under different genetic models were examined by multivariate logistic regression analysis, adjusting for confounders including age, sex, BMI and smoking history. Results were reported as odds ratios (ORs) with 95% confidence intervals (95% CI). Linkage disequilibrium (LD) was calculated using the SHEsis online software (http://analysis.bio-x.cn).

The meta-analyses were performed with STATA version 12.0 (StataCorp, College Station, Texas). The chi-squared-based *Q*-test and *I*-squared (*I*^2^) test were used to assess the between-study heterogeneity. A *P*-value of <0.10 or an *I*^2^-value of >50% suggested a statistically significant heterogeneity. A fixed-effect model was used for non-heterogeneous studies (Mantel–Haenszel method) and a random-effect model was used for heterogeneous studies (M–H heterogeneity method). Publication bias was evaluated by Begg’s funnel plot and Egger’s regression test. Hardy–Weinberg equilibrium (HWE) was tested in the control group for each study by the Chi-squared test. Statistical significance was defined as *P*<0.05 except the between-study heterogeneity analysis.

## Results

### Subject demographics

Baseline characteristics of the 410 cases and 418 controls are presented in Table 1. There were no statistical differences in demographic data including age, sex, smoking history and BMI. The case group consisted of 159 males and 251 females while 162 males and 256 females were in the control group. The average age of asthma onset in the cases was 33.69±14.26 years. The mean FEV1PP and FEV1/FVC % in the case group were 81.36±23.99 and 71.38±14.85, respectively.

**Table 1:** Characteristics of cases and controls

Characteristic	Case (*N* = 410)	Control (*N* = 418)	*P* value
Age (mean±SD)	44.02±13.77	44.09±13.75	0.944
Male *n* (%)	159 (38.78)	162 (38.76)	0.994
BMI (mean±SD)	23.06±3.19	22.95±3.34	0.634
Smoking history *n* (%)	76/403* (18.86)	55/262* (20.99)	0.58
Age of asthma onset (mean±SD)	33.69±14.26	–	–
FEV1PP (mean±SD)	81.36±23.99	–	–
FEV1/FVC % (mean±SD)	71.38±14.85	–	–
FVCPP (mean±SD)	95.63±10.18	–	–

* The number of participants providing smoking history.

Abbreviations: BMI, body mass index; FEV1PP, forced expiratory volume in one second expressed as percent of predicted; FVCPP, forced vital capacity expressed as percent of predicted.

### *CHI3L1* SNPs association study

**Figure 1:**
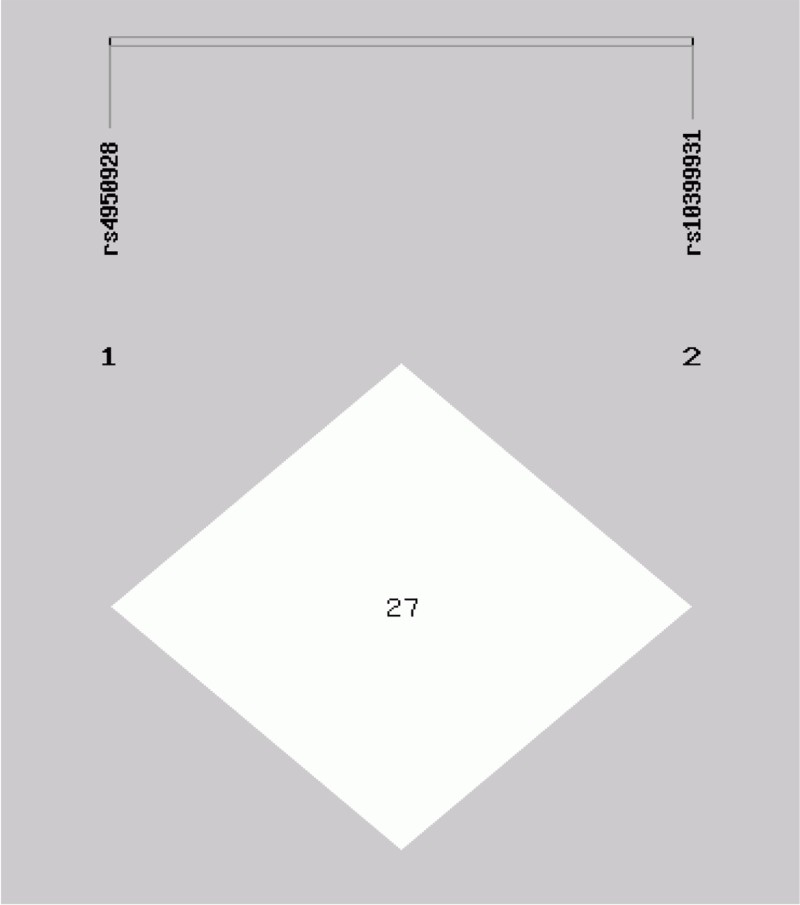
Analysis of linkage disequilibrium of rs4950928 and rs10399931

All subjects were successfully genotyped and no deviation from Hardy–Weinberg equilibrium was observed. The association results are presented in Table 2. After adjusting for confounding factors including age, sex, BMI and smoking history, the rs10399931 genotypes CT/TT were associated with increased risk of asthma under the dominant model (*P* = 0.031, OR = 1.428, 95% CI, 1.033–1.974) and genotype CT was associated with increased risk of asthma under the heterozygous model (*P* = 0.003, OR = 1.680, 95% CI, 1.186–2.380). No significant association was found between the rs4950928 and asthma under different genetic models. The LD between rs4950928 and rs10399931 was 0.27 (Figure 1). There was no significant difference observed in the haplotype analysis between the case and control groups (*P>*0.05) (Table 3).

**Table 2:** *CHI3L1* polymorphisms in cases and controls

SNPs	Cases *N* (%)	Controls *N* (%)	Genetic model^†^	OR (95%CI)	P^*^	Genetic model^†^	OR (95%CI)	P^*^
rs4950928(C>G)	410	418						
CC	305 (0.744)	303 (0.725)	Add	1.053 (0.770–1.438)	0.748	Hom	1.041 (0.916–1.182)	0.542
CG	94 (0.229)	101 (0.242)	Dom	1.098 (0.761–1.583)	0.616	Het	1.023 (0.792–1.322)	0.860
GG	11 (0.027)	14 (0.033)	Rec	0.857 (0.337–2.182)	0.746	All	1.107 (0.844–1.453)	0.462
rs10399931(C>T)	410	418						
CC	157 (0.383)	185 (0.443)	Add	1.088 (0.859–1.379)	0.484	Hom	0.831 (0.502–1.374)	0.471
CT	210 (0.512)	173 (0.414)	**Dom**	**1.428 (1.033–1.974)**	**0.031**	**Het**	**1.680 (1.186–2.380)**	**0.003**
TT	43 (0.105)	60 (0.144)	Rec	0.655 (0.410–1.048)	0.078	All	0.955 (0.781–1.168)	0.656

* Adjusted for sex, age, BMI and smoking history with logistic regression.

† All, allelic model; Add, additive model; Dom, dominant model; Rec, recessive model; Hom, homozygote model; Het, heterozygote model.

Values in bold: Rs10399931 was associated with increased risk of asthma under the dominant and heterozygous models.

**Table 3:** Haplotype analysis of *CHI3L1* in cases and controls

Haplotype	Cases *N* (%)	Controls *N* (%)	x^2^	P^*^	OR (95%CI)
CC	514.95 (0.628)	541.79 (0.648)	0.352	0.553	0.941 [0.769–1.151]
CT	189.05 (0.231)	165.21 (0.198)	3.020	0.082	1.232 [0.973–1.559]
GT	106.95 (0.130)	127.79 (0.153)	1.512	0.219	0.840 [0.637–1.109]
All others^†^	9.05 (0.011)	1.21 (0.001)	–	–	–

For each haplotype, alleles were arranged in order of rs4950928–rs10399931.

* Adjusted for sex, age, BMI and smoking history with logistic regression.

^†^The lowest frequency threshold (LFT) < 0.03 were pooled in this part.

### Functional analysis

**Figure 2:**
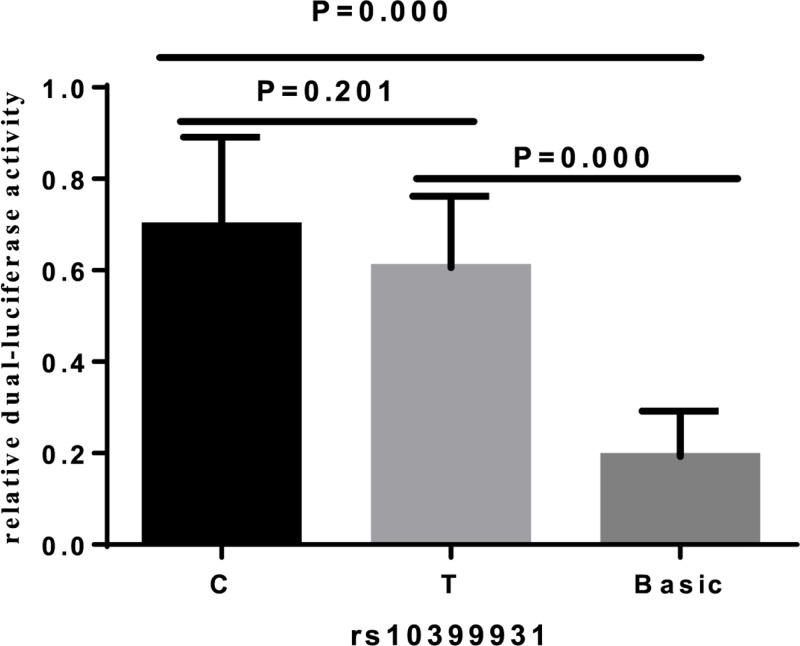
The allele dependent dual-luciferase activity expression of *CHI3L1* rs10399931 in HEK293 cells C, PGL3-basic-C; T, PGL3-basic-T; Basic, PGL3-basic.

**Figure 3:**
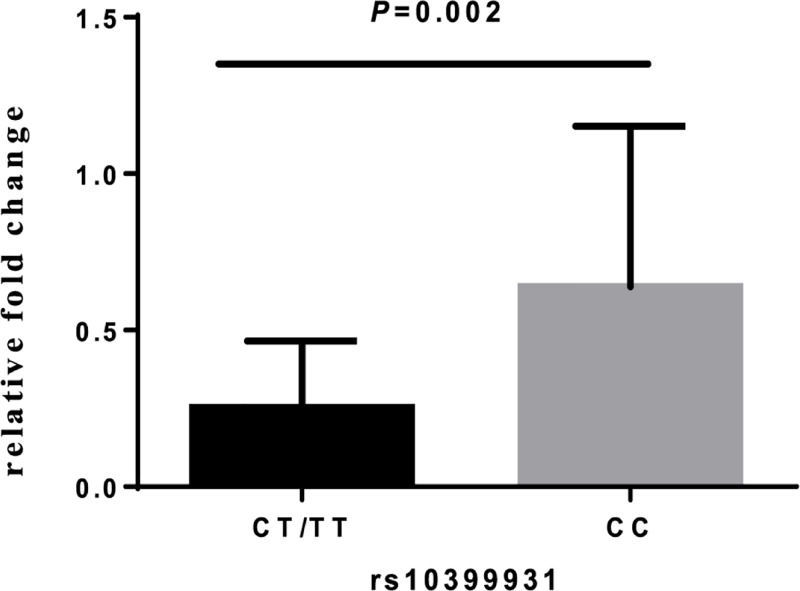
*CHI3L1* mRNA expression correlated with rs10399931

The JASPAR and F-SNP database were used to predict the potential function of asthma-susceptibility SNPs. rs10399931, located in the 5′-untranslated region of *CHI3L1*, was predicted to be a possible functional SNP with regulatory effects on gene transcription. The luciferase activity of rs10399931 allele C was higher than allele T in the dual-luciferase reporter gene analysis. However, there was no statistically significant difference between the two alleles (*P* = 0.201) (Figure 2). Compared with the CC genotype, the rs10399931 CT/TT genotypes reduced the relative mRNA expression detected by the real-time quantitative PCR method (*P* = 0.002) (Figure 3).

### Meta-analysis

We only successfully performed a meta-analysis of the relationship between rs4950928 and asthma due to the lack of association studies of other variants, including rs10399931, in *CHI3L1* with asthma. A total of 95 articles were identified in the initial searches of the above databases. Finally, 10 case–control studies from 9 published articles [3,6–13] and our current study containing 3519 cases and 7606 controls were identified in this meta-analysis, after a manual literature search based on the criteria (Supplementary Figure S1). The characteristics of each study are presented in Table 4.

**Table 4:** Main characteristics of all eligible studies in the meta-analysis

Author	Year	Ethnicity	Country	Populations	Case			Control			Method	*P* for HWE^*^
rs4950928					CC	CG	GG	CC	CG	GG		
Hansen et al. [6]	2015	Caucasian	Denmark	Adults	680	394	44	167	87	8	Centaurus Nanogen	0.406
James et al. [7]	2016	Caucasian	Sweden	Adults	69	34	8	40	14	3	TaqMan	0.254
Ramphul et al. [8]	2015	African	Mauritius	Children	122	64	6	121	63	5	TaqMan	0.339
LI et al. [9]	2015	Asian	China	Children	192	111	13	217	71	9	Mass Array-IPLEX	0.288
Rathcke et al. [10]	2009	Caucasian	Denmark	Adults	343	144	30	3495	1803	228	TaqMan	0.813
Ober et al. [3]	2008	Caucasian	USA	Children	227	100	17	150	120	24	TaqMan	1.000
Ober et al. [3]	2008	Caucasian	USA	Mixed	69	25	5	111	80	7	TaqMan	0.103
Shao [11]	2011	Asian	China	Adults	169	80	6	197	61	5	Mass Array-IPLEX	0.912
Naglot et al. [12]	2015	Asian	North India	Adults	0	32	68	0	19	31	PCR	0.097
Shao [13]	2017	Asian	China	Children	4	40	24	12	26	14	Mass Array-IPLEX	0.991
Chen et al.	2019	Asian	China	Adults	305	94	11	303	101	14	SNPscan™	0.129

* Hardy–Weinberg equilibrium (HWE) test was calculated in control group for each study.

In the overall meta-analysis, there was no statistically significant association between the *CHI3L1* rs4950928 polymorphism and the risk of asthma for all genetic models. Furthermore, subgroup analyses based on ethnicity and age were performed. Although the *P* value of the association between rs4950928 polymorphism and asthma was 0.046 among Asians under the allele model, it was close to 0.05 and *I*^2^ was >50% indicating a large heterogeneity detected in the result. Therefore, the result was not convincing and we did not consider that the rs4950928 variant contributed to the risk of asthma among Asians. Moreover, there was no significant association in the other subgroups (Table 5, Supplementary Figures S2–S4). There was no significant publication bias for the meta-analysis in all the genetic models (*P* > 0.05) (Table 6, Supplementary Figure S5).

**Table 5:** Meta-analysis of CHI3L1 polymorphism and asthma

Subgroup rs4950928	Genetic model	Genotype/allele	Heterogeneity*		Test of association		
			*I*^2^	*P*_het_	OR	95% CI	*P*_meta_
Overall	Recessive model	GG vs. GC + CC	0.000	0.705	1.18	0.94, 1.48	0.155
	Dominant model	GG + GC vs. CC	0.801	0.000	1.05	0.81, 1.37	0.696
	Allele model	G vs. C	0.733	0.000	0.94	0.78, 1.14	0.534
	Codominant model	GC vs. CC	0.794	0.000	1.04	0.79, 1.35	0.798
	Codominant model	GG vs. CC	0.392	0.097	1.17	0.91, 1.49	0.221
Caucasians	Recessive model	GG vs. GC + CC	0.308	0.216	1.16	0.86, 1.55	0.329
	Dominant model	GG + GC vs. CC	0.764	0.002	0.82	0.60, 1.13	0.221
	Allele model	G vs. C	0.755	0.003	0.89	0.68, 1.15	0.363
	Codominant model	GC vs. CC	0.732	0.005	0.79	0.58, 1.08	0.142
	Codominant model	GG vs. CC	0.500	0.091	1.07	0.80, 1.44	0.648
Asians	Recessive model	GG vs. GC + CC	0.000	0.839	1.21	0.84, 1.75	0.313
	Dominant model	GG + GC vs. CC	0.774	0.004	1.52	0.97, 2.40	0.070
	Allele model	G vs. C	0.584	0.047	1.30	1.00, 1.69	0.046
	Codominant model	GC vs. CC	0.743	0.009	1.52	0.97, 2.36	0.065
	Codominant model	GG vs. CC	0.494	0.115	1.45	0.90, 2.33	0.131
Children	Recessive model	GG vs. GC + CC	0.288	0.239	0.98	0.66, 1.46	0.915
	Dominant model	GG + GC vs. CC	0.905	0.000	1.28	0.62, 2.61	0.506
	Allele model	G vs. C	0.891	0.000	1.12	0.67, 1.86	0.678
	Codominant model	GC vs. CC	0.892	0.000	1.26	0.63, 2.55	0.515
	Codominant model	GG vs. CC	0.760	0.006	1.02	0.66, 1.57	0.938
Adults	Recessive model	GG vs. GC + CC	0.000	0.890	1.28	0.97, 1.69	0.086
	Dominant model	GG + GC vs. CC	0.544	0.067	1.07	0.87, 1.32	0.531
	Allele model	G vs. C	0.261	0.238	1.07	0.93, 1.23	0.365
	Codominant model	GC vs. CC	0.600	0.041	1.06	0.83, 1.34	0.654
	Codominant model	GG vs. CC	0.000	0.810	1.25	0.92, 1.70	0.151

* Test for heterogeneity: Random-effects model was used when *P* value for heterogeneity test <0.10 and *I*^2^>50%; otherwise, fixed-effects model was used. Abbreviations: CI, confidence interval; OR, odds ratio; *P*_het_, *P*-value of heterogeneity test; *P*_meta_, *P*-value of pooled effect.

**Table 6:** Results of publication bias test

rs4950928	Genetic model	Genotype/allele	Begg’s test		Egger’s test	
			*z* value	*P* value	*t* value	*P* value
Overall	Recessive model	GG vs. GC + CC	0.620	0.533	−0.220	0.832
	Dominant model	GG + GC vs. CC	0.720	0.474	1.080	0.310
	Allele model	G vs. C	0.470	0.640	0.950	0.366
	Codominant model	GC vs. CC	0.720	0.474	1.110	0.301
	Codominant model	GG vs. CC	0.720	0.474	0.630	0.543

## Discussion

In this present study, we investigated the association between asthma and two common SNPs (rs10399931 and rs4950928) of *CHI3L1* in the southwest Chinese Han population and tried to identify the influence of rs10399391 on the allelic expression of *CHI3L1*. The *CHI3L1* gene spans 7948bp with 10 exons in the human genome [14] and is a susceptibility locus for many diseases including cancer, autoimmune diseases, schizophrenia and chronic inflammatory diseases including asthma and chronic obstructive pulmonary disease [15].

As promoter SNPs of *CHI3L1*, rs4950928 and rs10399931 were frequently reported in many association studies of *CHI3L1* genetic polymorphisms and asthma. However, the results in different populations were inconsistent and controversial. Ober et al. [3] found that the rs4950928 allele C was associated with elevated serumYKL-40 protein levels, bronchial hyper-responsiveness and reduced lung function in the Hutterites. Verlaan et al. [16] revealed the same positive association between the allele C of rs4950928 and increased *CHI3L1* expression in Caucasian and Yoruban African asthmatic individuals. James et al. [17] found that the *CHI3L1* rs4950928 CC genotype was associated with higher YKL-40 levels than the GG genotype in preschool children with wheeze. Interestingly, Rathcke et al. [10] published an opposite result that the rs4950928 allele G, not allele C, was associated with asthma in 6514 Danish asthmatic adults. Li et al. [9] reported the rs4950928 allele G increased the risk of Chinese childhood asthma. In addition, Hansen et al. [6] reported no association between polymorphisms of rs4950928 and asthma in 1921 subjects from Denmark. Rs4950928 had little contribution to the development of childhood asthma in the Mauritian population [8] and to atopy in Korean children [18], which was a similar result to that of James et al. [7] in European people. Gomez et al. [19] did not find association of the polymorphism with airflow obstruction or airway levels of YKL-40 in asthmatic individuals of either European or African ancestry.

As for rs10399931, there were also inconsistent results in a variety of studies. Verlaan et al. [16] confirmed that rs10399931 was functional and the C allele increased gene expression in Caucasian and Yoruban African asthmatic individuals. The authors concluded that rs4950928 and rs10399931 were equally likely to mediate susceptibility to asthma due to their strong LD structure. Furthermore, rs10399931 appeared to be significantly associated with FEV1/FVC ratio in the report of Rathcke et al. [10]. Tsai et al. [20] reported that the rs10399931GG genotype was associated with elevated serumYKL-40 levels and severity of lung obstruction in steroid-using asthma patients from southern Taiwan. By contrast, Sohn et al. [18] did not observe any significant association between rs10399931 and atopy in Korean children. Usemann et al. [21] failed to identify a correlation of rs10399931 and YKL-40 with atopy and lung function in 6 years old children after correction for multiple testing. No association was observed in asthmatic individuals of African ancestry [19], either.

In our study, we did not find an association between rs4950928 and asthma. The rs10399931 genotypes CT/TT were associated with increased risk of asthma under the dominant model and there was a similar association with genotype CT under the heterozygous model. Although the rs10399931 T and C alleles increased the luciferase activity, there was no significant difference between the two alleles. The relative mRNA expression of the rs10399931CC genotype was higher than CT/TT genotypes. Parts of the above results were consistent with some previous studies. We speculate the reasons for this discrepancy as follows: Firstly, the allele and genotype frequency, as well as LD between rs4950928 and rs10399931 differ in different ethnic populations. This provides a potential reason that results may not always replicate in other populations. Asthma is caused by the interaction of environmental and genetic factors and there are several asthma phenotypes. Therefore, even though the subjects were from the same ethnic group, different environmental exposures, geographical distributions and habits may lead to diversity in the pathogenesis and regulation of disease. In the study by Tsai et al. [20], the ethnicity of the participants was not described in detail. In fact, there are many minorities in Taiwan which are different from the Chinese mainland. That may explain the different results between the present study and the ones from Taiwan [20] and Korea [18]. Secondly, different protocols or design of studies such as inclusion and exclusion criteria of subjects may lead to different results. The asthma patients in our study were younger with worse pulmonary function than the ones in the study of Tsai et al. [20]. In addition, our study was focused on late-onset adult asthma (as reflected in the mean age of onset), and therefore may be expected to generate different results to previous studies that included only children with asthma. Lastly, replicated studies with larger sample size in Chinese Han are warranted to enhance the reliability of results, although the power calculation indicated that the present study has adequate power to detect small to moderate effect sizes.

In addition, due to the ambiguous results of different studies on the relationship between *CHI3L1* polymorphisms and asthma, a meta-analysis was performed using data collected from previously published reports and the present study. Unfortunately, only the rs4950928 meta-analysis was successfully conducted. Analysis of the other variants was not performed, as the number of the published articles was too small. We failed to find any association between rs4950928 and asthma in the overall analysis as well as in the stratification analysis, which was consistent with the current study. Due to the high heterogeneity of the relation between rs4950928 and asthma in the Asians under the allele model, the result was unreliable. Therefore, further meta-analysis is needed to include more eligible articles published in the future. This was an updated meta-analysis drawing the same conclusion as the report by Xu et al. [22], one of the two published meta-analyses [23]. The other meta-analysis was performed by Zhu et al. [23] but only included four studies, which may result in different results.

This was the first association study between *CHI3L1* and asthma in the southwest Chinese Han population and there are some limitations. As the effect of each single polymorphism on asthma susceptibility was weak, we did not correct the results for multiple testing to due to its conservative nature. Furthermore, it would have been better to include more SNPs of *CHI3L1* to be tested in this association study, including rs12141494 [19] and rs10399805 [18]. Furthermore, there was a lack of measurement of serum YKL-40 levels.

In conclusion, *CHI3L1* was a susceptibility locus for asthma in the southwest Chinese Han population and rs10399931 may be a functional variant of *CHI3L1*. The rs10399931 CT/TT genotypes may increase the risk of asthma and reduce the relative mRNA expression. There was no significant difference between allele C and T in the transcriptional regulation activity.

## Supporting information

**Supplementary Figure S1:** Flowchart of the literature search

**Supplementary Figure S2:** Forest plot of the association between *CHI3L1* rs4950928 and asthma under different models. a, the recessive model; b, dominant model; c, codominant (GC vs. CC); d, codominant (GG vs. CC); e, allele model.

**Supplementary Figure S3:** Forest plot of the association between *CHI3L1* rs4950928 and asthma by ethnicity stratification under different models. a, the recessive model; b, dominant model; c, codominant (GC vs. CC); d, codominant (GG vs. CC); e, allele model.

**Supplementary Figure S4:** Forest plot of the association between *CHI3L1* rs4950928 and asthma by age stratification under different models. a, the recessive model; b, dominant model; c, codominant (GC vs. CC); d, codominant (GG vs. CC); e, allele model.

**Supplementary Figure S5:** Begg’s funnel plots of publication bias for all studies a, recessive model; b, dominant model; c, codominant (GC vs. CC); d, codominant (GG vs. CC); e, allele model.

## Abbreviations

CHI3L1: chitinase 3-like 1
FEV1: forced expiratory volume in the first second
FVC: forced vital capacity
GWAS: genome-wide association study
HWE: Hardy–Weinberg equilibrium
LD: linkage disequilibrium
SNP: single nucleotide polymorphism

## References

[1] Masoli M., Fabian D., Holt S. and Beasley R., Global Initiative for Asthma Program(2004) The global burden of asthma: executive summary of the GINA Dissemination Committee report. Allergy 59, 469–478 10.1111/j.1398-9995.2004.00526.x 15080825

[2] Asthma. GIf (2015) GINA Global Strategy for Asthma Management and Prevention updated. http://www.ginasthma.org

[3] Ober C., Tan Z., Sun Y., Possick J.D., Pan L., Nicolae R. (2008) Effect of variation in CHI3L1 on serum YKL-40 level, risk of asthma, and lung function. N. Engl. J. Med. 358, 1682–1691 10.1056/NEJMoa0708801 18403759 PMC2629486

[4] Lee C.G., Hartl D., Lee G.R., Koller B., Matsuura H., Da Silva C.A. (2009) Role of breast regression protein 39 (BRP-39)/chitinase 3-like-1 in Th2 and IL-13-induced tissue responses and apoptosis. J. Exp. Med. 206, 1149–1166 10.1084/jem.20081271 19414556 PMC2715037

[5] Johansen J.S. (2006) Studies on serum YKL-40 as a biomarker in diseases with inflammation, tissue remodelling, fibroses and cancer. Dan. Med. Bull. 53, 172–209 17087877

[6] Hansen J.W., Thomsen S.F., Porsbjerg C., Rasmussen L.M., Harmsen L., Johansen J.S. (2015) YKL-40 and genetic status of CHI3L1 in a large group of asthmatics. Eur. Clin. Respir. J. 2, 25117 10.3402/ecrj.v2.25117 26672955 PMC4653313

[7] James A.J., Reinius L.E., Verhoek M., Gomes A., Kupczyk M., Hammar U. (2016) Increased YKL-40 and chitotriosidase in asthma and chronic obstructive pulmonary disease. Am. J. Respir. Crit. Care Med. 193, 131–142 10.1164/rccm.201504-0760OC 26372680

[8] Ramphul K., Hua L., Bao Y.X., Li J.Y., Liu Q.H., Ji R.X. (2015) Identification of IL13 C1923T as a single nucleotide polymorphism for asthma in children from Mauritius. Pediatr. Allergy Immunol. Pulmonol. 28, 92–95 10.1089/ped.2014.0464 26155367 PMC4491167

[9] Li J.M., Zhang H.F., Shen X.L., Xie H., Wu X.D., Shen T. (2015) Association between CHI3L1 SNPs and susceptibility to childhood asthma. Zhongguo dang dai er ke za zhi = Chin. J. Contemp. Pediatr. 17, 144–148 25760838

[10] Rathcke C.N., Holmkvist J., Husmoen L.L., Hansen T., Pedersen O., Vestergaard H. (2009) Association of polymorphisms of the CHI3L1 gene with asthma and atopy: a populations-based study of 6514 Danish adults. PLoS One 4, e6106 10.1371/journal.pone.0006106 19568425 PMC2699472

[11] JL S. (2011) Effect of Variation in CHI3L1 on Plasma YKL-40 Level, Risk of Asthma, and Lung Function, Southern Medical University

[12] Naglot S., Dalal K., Aggarwal P. and Dada R. (2015) Association of CG genotype at rs4950928 promoter in CHI3L1 gene with YKL-40 levels and asthma susceptibility in North Indian asthma patients. Indian J. Clin. Biochem. 30, 403–411 10.1007/s12291-015-0478-0

[13] YW S. (2017) Association Research Between CHI3L1 Polymorphism of Asthma Susceptibility Gene and Airway Remodeling and Inflammatory Response, Lanzhou University

[14] Johansen J.S., Hvolris J., Hansen M., Backer V., Lorenzen I. and Price P.A. (1996) Serum YKL-40 levels in healthy children and adults. Comparison with serum and synovial fluid levels of YKL-40 in patients with osteoarthritis or trauma of the knee joint. Br. J. Rheumatol. 35, 553–559 10.1093/rheumatology/35.6.553 8670576

[15] Coffman F.D. (2008) Chitinase 3-like-1 (CHI3L1): a putative disease marker at the interface of proteomics and glycomics. Crit. Rev. Clin. Lab. Sci. 45, 531–562 10.1080/10408360802334743 19003601

[16] Verlaan D.J., Ouimet M., Adoue V., Sirois-Gagnon D., Lariviere M., Ge B. (2012) Promoter polymorphisms in CHI3L1 are associated with asthma. J. Allergy Clin. Immunol. 130, 533–535 10.1016/j.jaci.2012.03.015 22534532

[17] James A., Stenberg Hammar K., Reinius L., Konradsen J.R., Dahlen S.E., Soderhall C. (2017) A longitudinal assessment of circulating YKL-40 levels in preschool children with wheeze. Pediatr. Allergy Immunol. 28, 79–85 10.1111/pai.12669 27732738

[18] Sohn M.H., Lee J.H., Kim K.W., Kim S.W., Lee S.H., Kim K.E. (2009) Genetic variation in the promoter region of chitinase 3-like 1 is associated with atopy. Am. J. Respir. Crit. Care Med. 179, 449–456 10.1164/rccm.200809-1422OC 19106306

[19] Gomez J.L., Crisafi G.M., Holm C.T., Meyers D.A., Hawkins G.A., Bleecker E.R. (2015) Genetic variation in chitinase 3-like 1 (CHI3L1) contributes to asthma severity and airway expression of YKL-40. J. Allergy Clin. Immunol. 136, 51.e10–58.e10 10.1016/j.jaci.2014.11.027 25592985 PMC4494869

[20] Tsai Y., Ko Y., Huang M., Lin M., Wu C., Wang C. (2014) CHI3L1 polymorphisms associate with asthma in a Taiwanese population. BMC Med. Genet. 15, 86 10.1186/1471-2350-15-86 25056157 PMC4113488

[21] Usemann J., Frey U., Mack I., Schmidt A., Gorlanova O., Roosli M. (2016) CHI3L1 polymorphisms, cord blood YKL-40 levels and later asthma development. BMC Pulmonary Med. 16, 81 10.1186/s12890-016-0239-8 27193312 PMC4870763

[22] Changdi Xu F.L., Wang Quan and Tang Heng (2017) Negative association between chitinase 3-Like-1 C-131G polymorphism and asthma-from a meta-analysis. Int. J. Clin. Exp. Med. 10, 8745–8752

[23] Zhu Y., Yan X., Zhai C., Yang L. and Li M. (2017) Association between risk of asthma and gene polymorphisms in CHI3L1 and CHIA: a systematic meta-analysis. BMC Pulmonary Med. 17, 193 10.1186/s12890-017-0515-2 29233108 PMC5726029

