# Supplementary material for: Functional study of the association of CHI3L1 polymorphisms with asthma susceptibility in the Southwest Chinese Han population
Source: Biosci Rep. 2019 May 14;39(5):BSR20182008. doi: 10.1042/BSR20182008 (PMC6522726; doi:10.1042/BSR20182008)
Supplement: Supplementary file 1 [file BSR-2018-2008_suppS1.pdf]

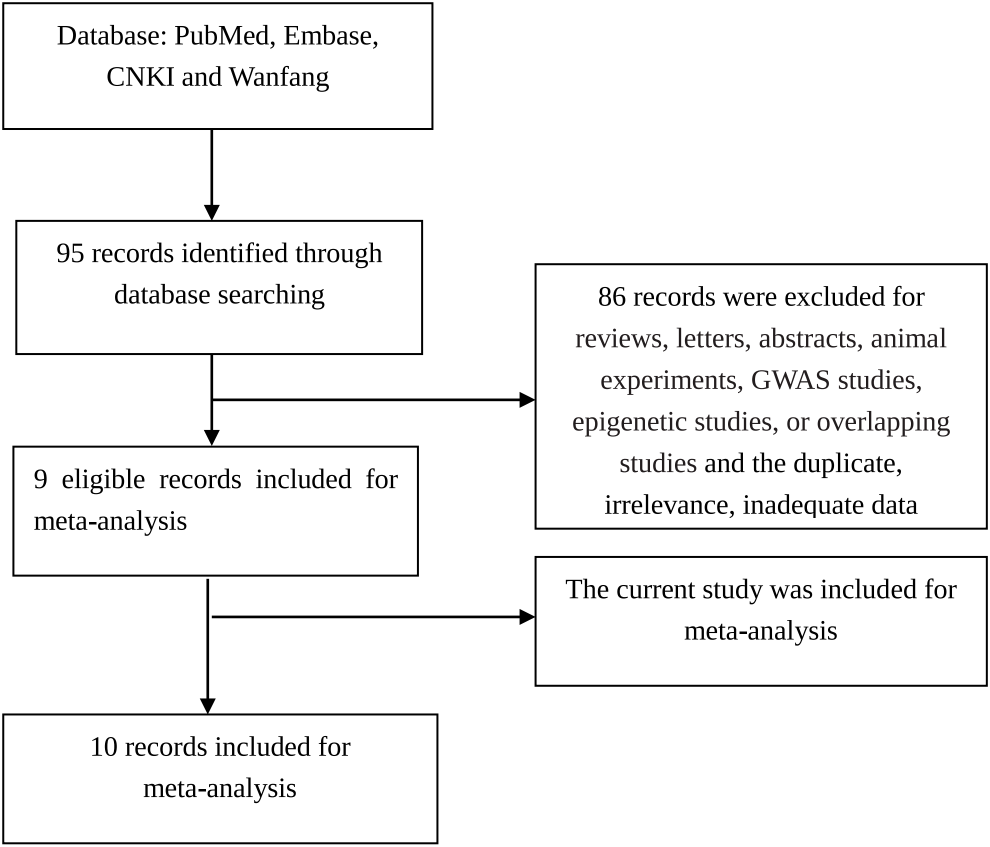

Figure S1 Flowchart of the literature search

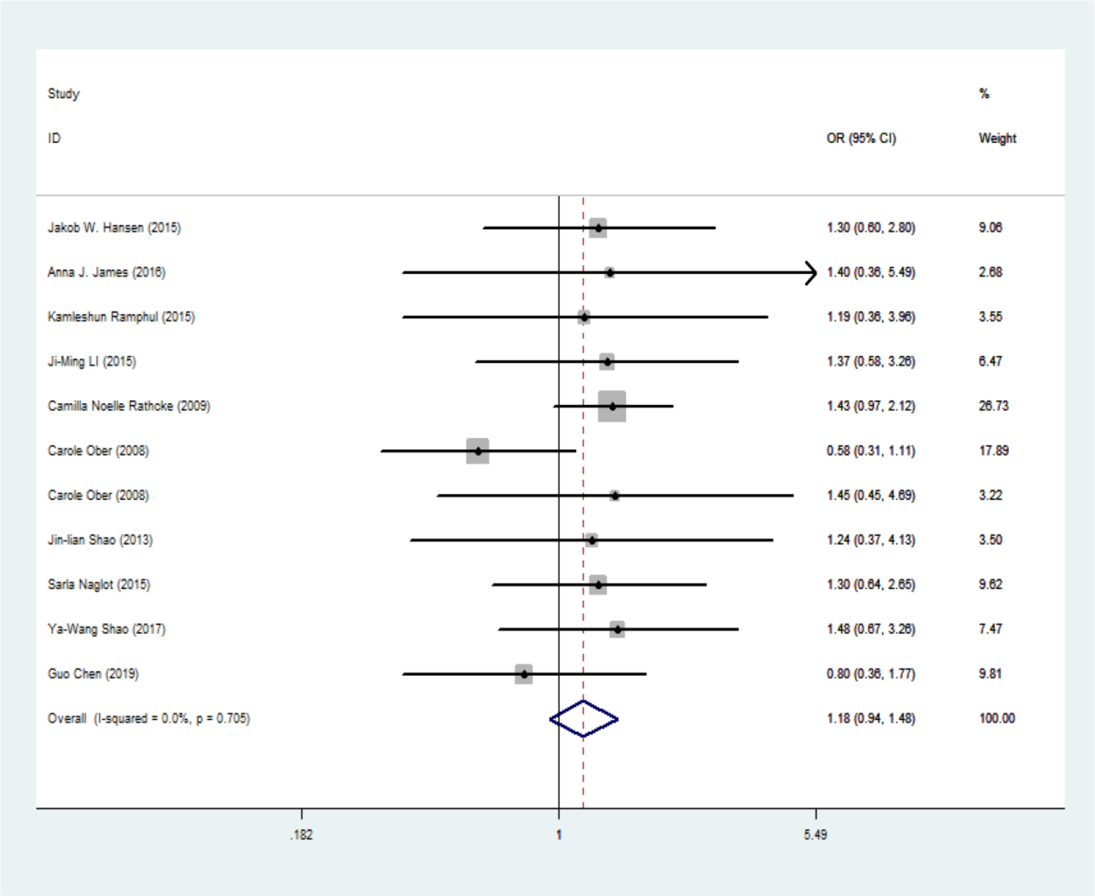
Study
ID
OR (95% CI)
%
Weight
Jakob W. Hansen (2015)
1.30 (0.60, 2.80)
9.06
Anna J. James (2016)
1.40 (0.36, 5.49)
2.68
Kamleshun Ramphul (2015)
1.19 (0.36, 3.96)
3.55
Ji-Ming LI (2015)
1.37 (0.58, 3.26)
6.47
Camilla Noelle Rathcke (2009)
1.43 (0.97, 2.12)
26.73
Carole Ober (2008)
0.58 (0.31, 1.11)
17.89
Carole Ober (2008)
1.45 (0.45, 4.69)
3.22
Jin-lian Shao (2013)
1.24 (0.37, 4.13)
3.50
Sarla Naglot (2015)
1.30 (0.64, 2.65)
9.62
Ya-Wang Shao (2017)
1.48 (0.67, 3.26)
7.47
Guo Chen (2019)
0.80 (0.36, 1.77)
9.81
Overall (I-squared = 0.0%, p = 0.705)
1.18 (0.94, 1.48)
100.00
.182
1
5.49

a

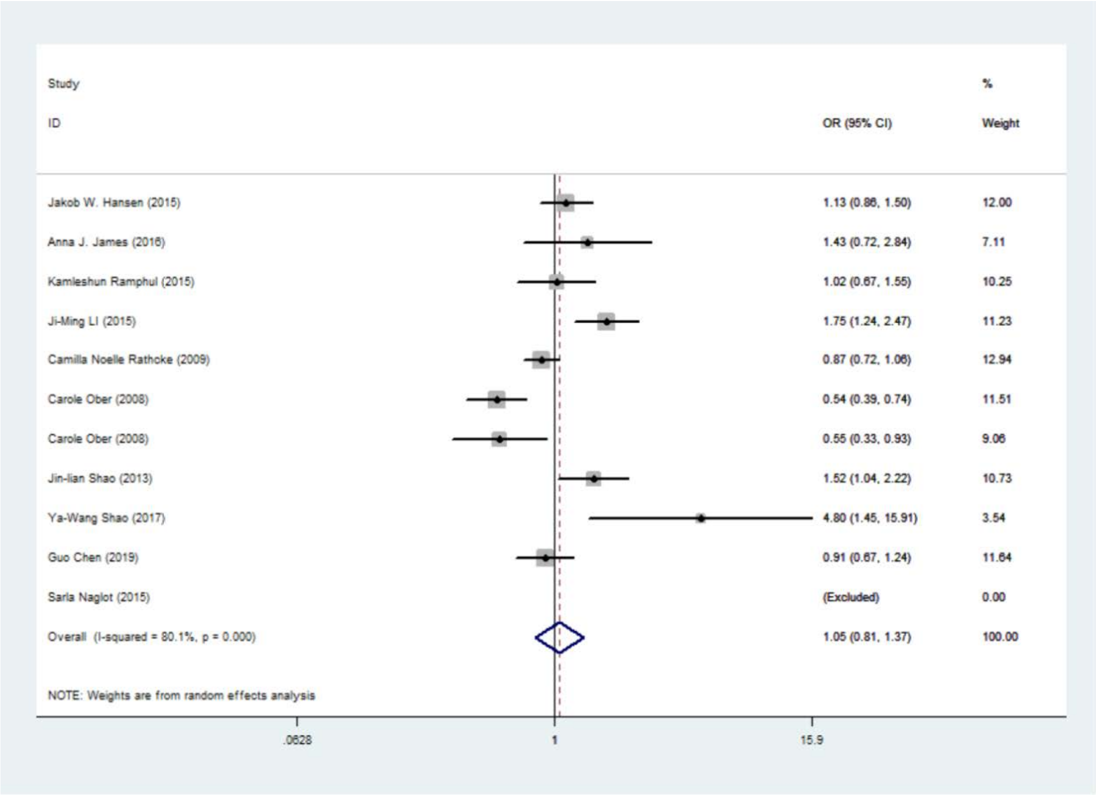
Study
ID
OR (95% CI)
%
Weight
Jakob W. Hansen (2015)
1.13 (0.86, 1.50)
12.00
Anna J. James (2016)
1.43 (0.72, 2.84)
7.11
Kamleshun Ramphul (2015)
1.02 (0.67, 1.55)
10.25
Ji-Ming LI (2015)
1.75 (1.24, 2.47)
11.23
Camilla Noelle Rathcke (2009)
0.87 (0.72, 1.06)
12.94
Carole Ober (2008)
0.54 (0.39, 0.74)
11.51
Carole Ober (2008)
0.55 (0.33, 0.93)
9.06
Jin-lian Shao (2013)
1.52 (1.04, 2.22)
10.73
Ya-Wang Shao (2017)
4.80 (1.45, 15.91)
3.54
Guo Chen (2019)
0.91 (0.67, 1.24)
11.64
Sarla Naglot (2015)
(Excluded)
0.00
Overall (I-squared = 80.1%, p = 0.000)
1.05 (0.81, 1.37)
100.00
NOTE: Weights are from random effects analysis
.0628
1
15.9

b

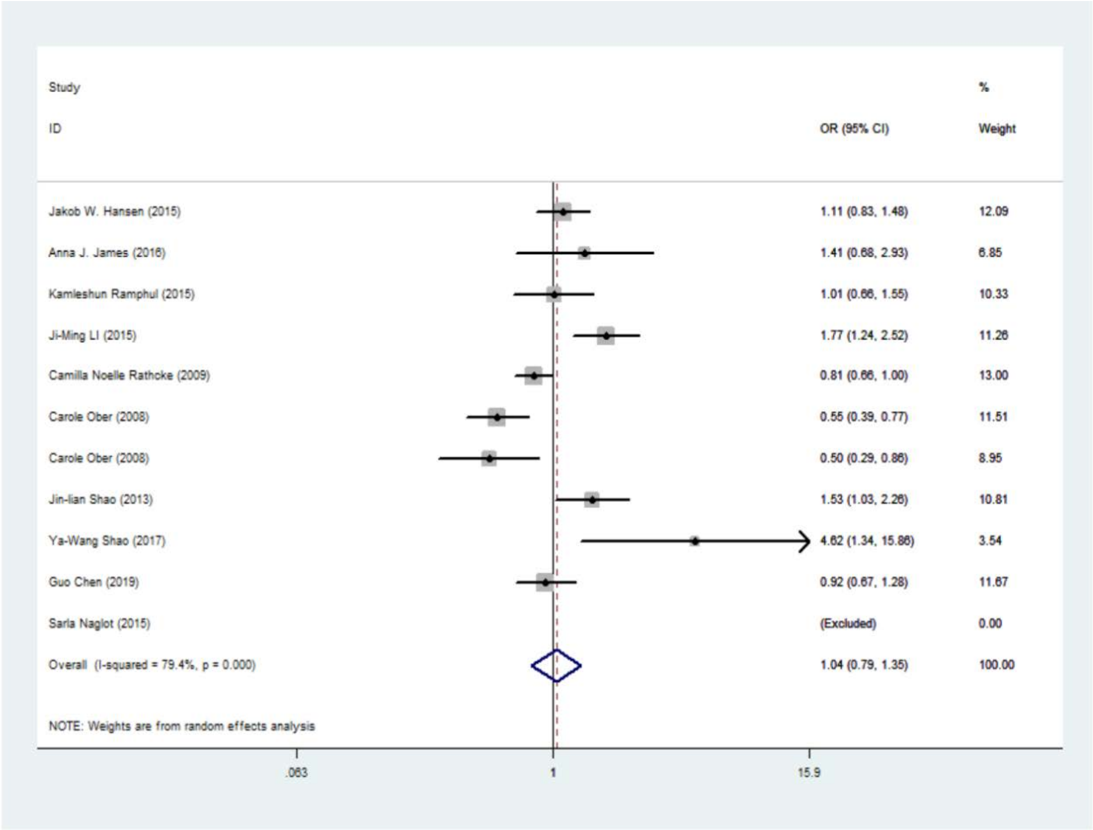
Study
ID
OR (95% CI)
%
Weight
Jakob W. Hansen (2015) 1.11 (0.83, 1.48) 12.09
Anna J. James (2016) 1.41 (0.68, 2.93) 6.85
Kamleshun Ramphul (2015) 1.01 (0.66, 1.55) 10.33
Ji-Ming LI (2015) 1.77 (1.24, 2.52) 11.26
Camilla Noelle Rathcke (2009) 0.81 (0.66, 1.00) 13.00
Carole Ober (2008) 0.55 (0.39, 0.77) 11.51
Carole Ober (2008) 0.50 (0.29, 0.86) 8.95
Jin-lian Shao (2013) 1.53 (1.03, 2.26) 10.81
Ya-Wang Shao (2017) 4.62 (1.34, 15.86) 3.54
Guo Chen (2019) 0.92 (0.67, 1.28) 11.67
Sarla Naglot (2015) (Excluded) 0.00
Overall (I-squared = 79.4%, p = 0.000) 1.04 (0.79, 1.35) 100.00
NOTE: Weights are from random effects analysis
.063
1
15.9

c

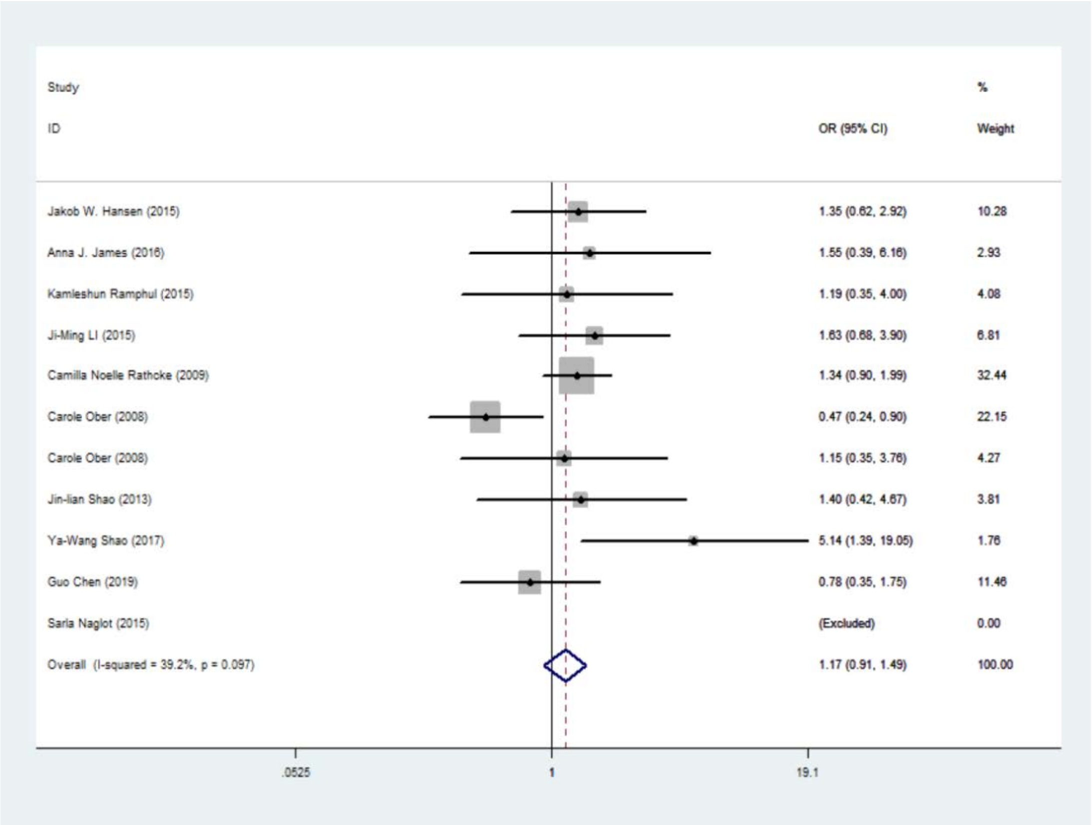
Study
ID
OR (95% CI)
%
Weight
Jakob W. Hansen (2015) 1.35 (0.62, 2.92) 10.28
Anna J. James (2016) 1.55 (0.39, 6.16) 2.93
Kamleshun Ramphul (2015) 1.19 (0.35, 4.00) 4.08
Ji-Ming LI (2015) 1.63 (0.68, 3.90) 6.81
Camilla Noelle Rathcke (2009) 1.34 (0.90, 1.99) 32.44
Carole Ober (2008) 0.47 (0.24, 0.90) 22.15
Carole Ober (2008) 1.15 (0.35, 3.76) 4.27
Jin-lian Shao (2013) 1.40 (0.42, 4.67) 3.81
Ya-Wang Shao (2017) 5.14 (1.39, 19.05) 1.76
Guo Chen (2019) 0.78 (0.35, 1.75) 11.46
Sarla Naglot (2015) (Excluded) 0.00
Overall (I-squared = 39.2%, p = 0.097) 1.17 (0.91, 1.49) 100.00
.0525
1
19.1

d

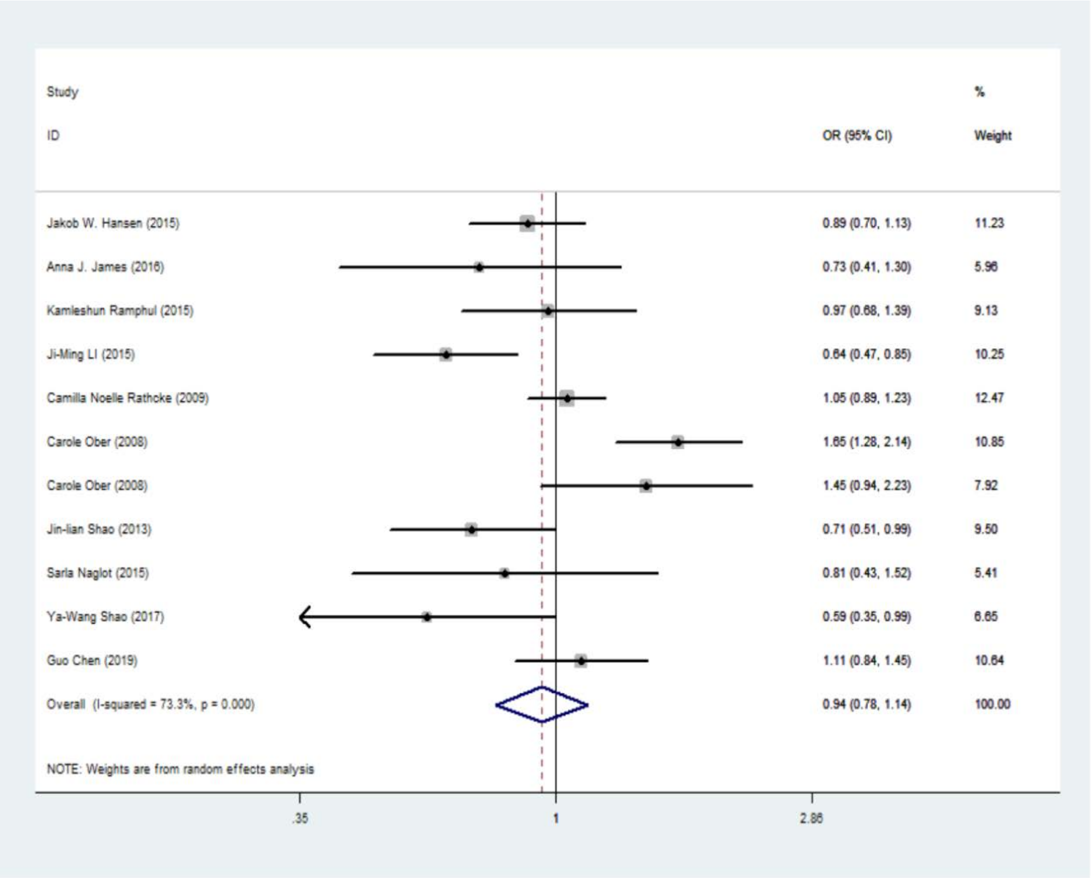

e

Figure S2 Forest plot of the association between *CHI3L1* rs4950928 and asthma under different models. a, the recessive model; b, dominant model; c, codominant (GC vs. CC); d, codominant (GG vs. CC); e, allele model.

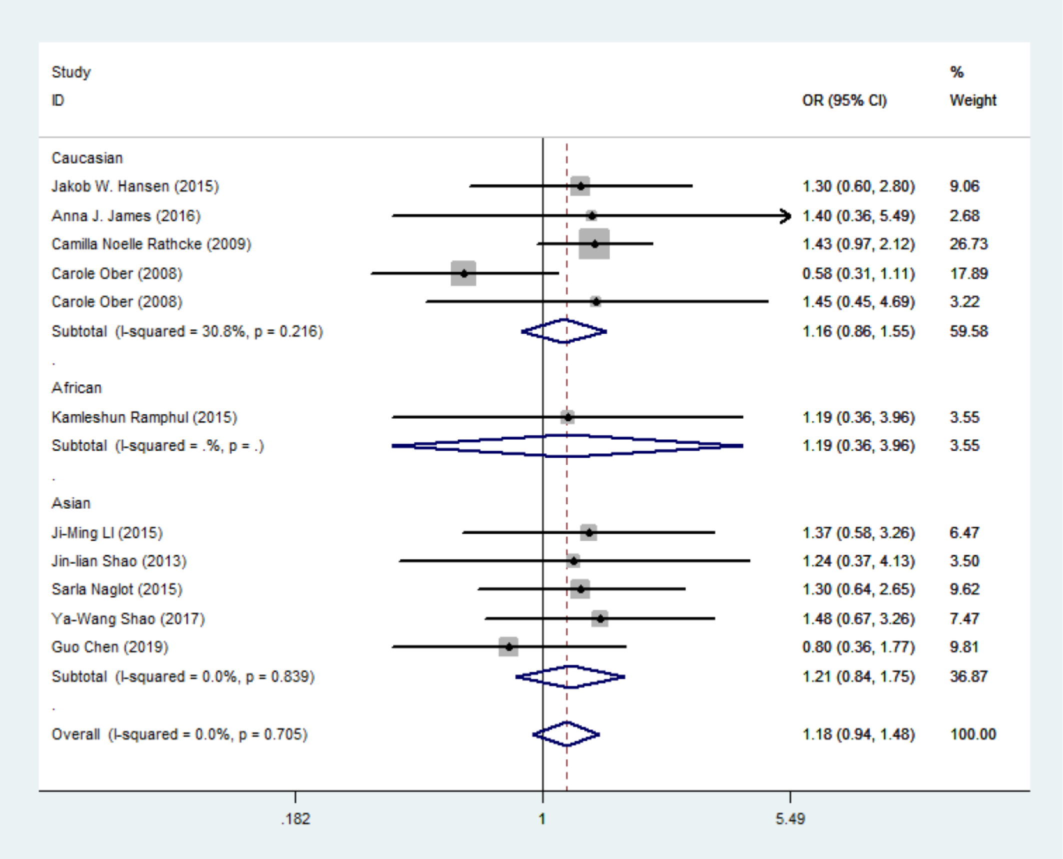
Study
ID
OR (95% CI)
%
Weight
Caucasian
Jakob W. Hansen (2015) 1.30 (0.60, 2.80) 9.06
Anna J. James (2016) 1.40 (0.36, 5.49) 2.68
Camilla Noelle Rathcke (2009) 1.43 (0.97, 2.12) 26.73
Carole Ober (2008) 0.58 (0.31, 1.11) 17.89
Carole Ober (2008) 1.45 (0.45, 4.69) 3.22
Subtotal (I-squared = 30.8%, p = 0.216) 1.16 (0.86, 1.55) 59.58
.
African
Kamleshun Ramphul (2015) 1.19 (0.36, 3.96) 3.55
Subtotal (I-squared = .%, p = .) 1.19 (0.36, 3.96) 3.55
.
Asian
Ji-Ming LI (2015) 1.37 (0.58, 3.26) 6.47
Jin-lian Shao (2013) 1.24 (0.37, 4.13) 3.50
Sarla Naglot (2015) 1.30 (0.64, 2.65) 9.62
Ya-Wang Shao (2017) 1.48 (0.67, 3.26) 7.47
Guo Chen (2019) 0.80 (0.36, 1.77) 9.81
Subtotal (I-squared = 0.0%, p = 0.839) 1.21 (0.84, 1.75) 36.87
.
Overall (I-squared = 0.0%, p = 0.705) 1.18 (0.94, 1.48) 100.00
.182
1
5.49

a

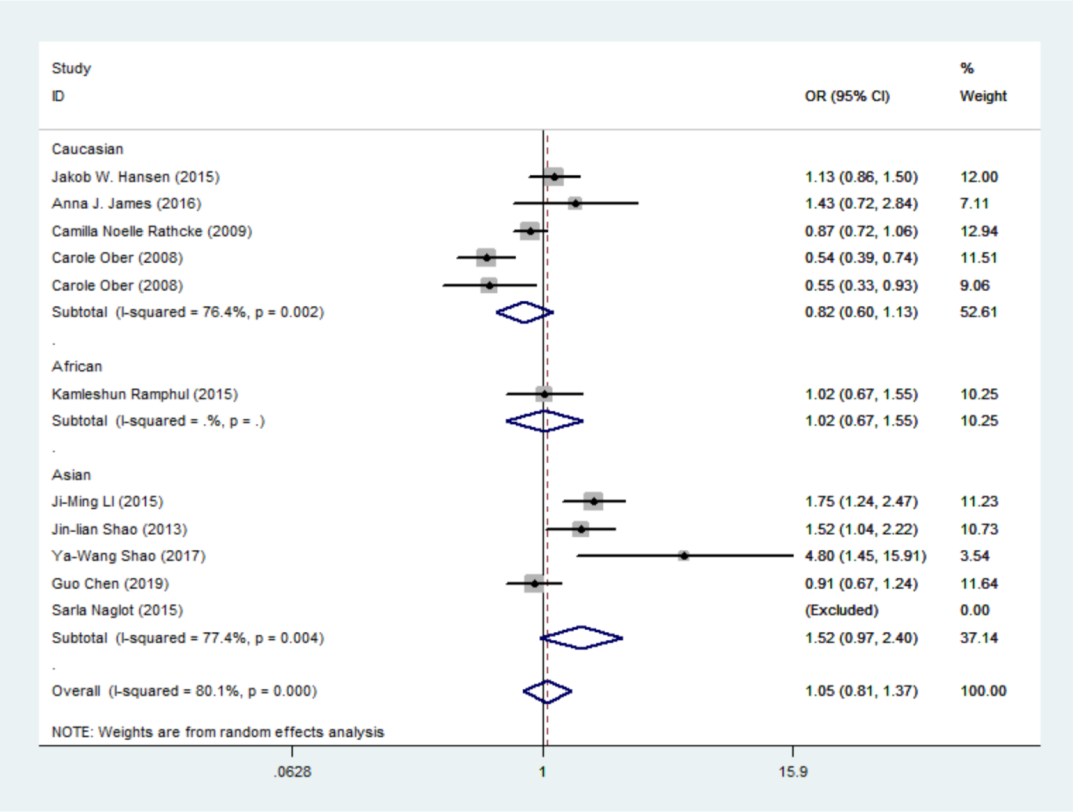
Study
ID
OR (95% CI)
%
Weight
Caucasian
Jakob W. Hansen (2015) 1.13 (0.86, 1.50) 12.00
Anna J. James (2016) 1.43 (0.72, 2.84) 7.11
Camilla Noelle Rathcke (2009) 0.87 (0.72, 1.06) 12.94
Carole Ober (2008) 0.54 (0.39, 0.74) 11.51
Carole Ober (2008) 0.55 (0.33, 0.93) 9.06
Subtotal (I-squared = 76.4%, p = 0.002) 0.82 (0.60, 1.13) 52.61
.
African
Kamleshun Ramphul (2015) 1.02 (0.67, 1.55) 10.25
Subtotal (I-squared = .%, p = .) 1.02 (0.67, 1.55) 10.25
.
Asian
Ji-Ming LI (2015) 1.75 (1.24, 2.47) 11.23
Jin-lian Shao (2013) 1.52 (1.04, 2.22) 10.73
Ya-Wang Shao (2017) 4.80 (1.45, 15.91) 3.54
Guo Chen (2019) 0.91 (0.67, 1.24) 11.64
Sarla Naglot (2015) (Excluded) 0.00
Subtotal (I-squared = 77.4%, p = 0.004) 1.52 (0.97, 2.40) 37.14
.
Overall (I-squared = 80.1%, p = 0.000) 1.05 (0.81, 1.37) 100.00
NOTE: Weights are from random effects analysis
.0628
1
15.9

b

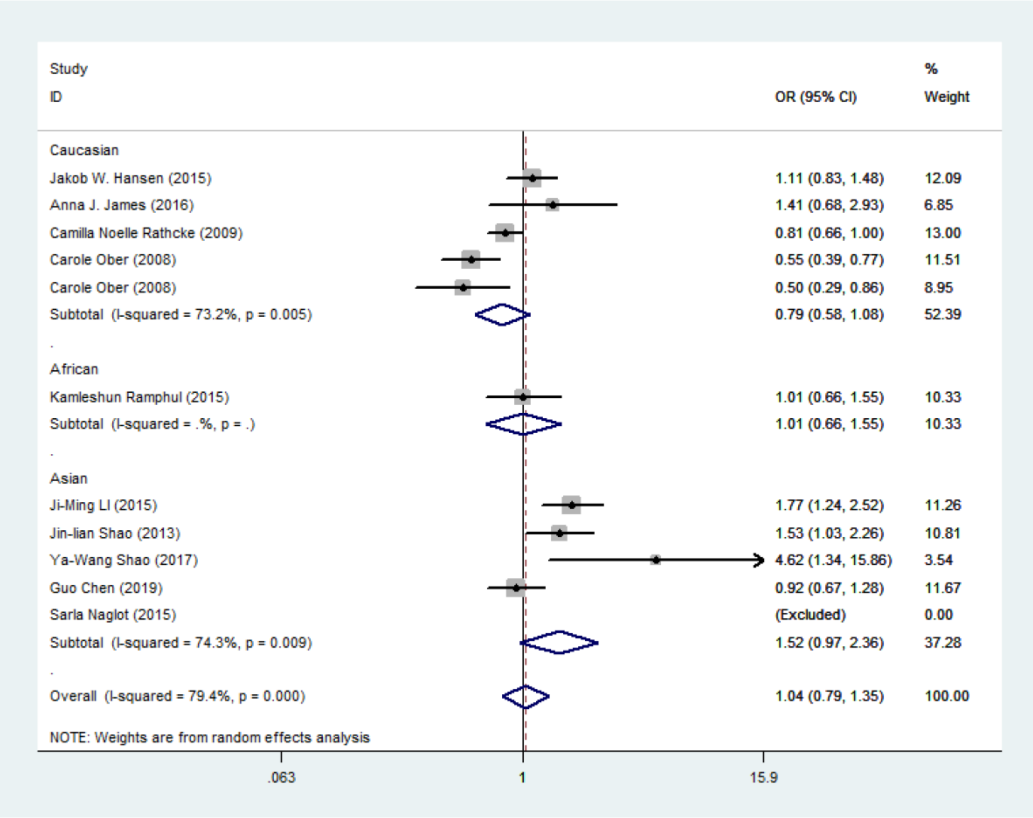
Study
ID
OR (95% CI)
%
Weight
Caucasian
Jakob W. Hansen (2015) 1.11 (0.83, 1.48) 12.09
Anna J. James (2016) 1.41 (0.68, 2.93) 6.85
Camilla Noelle Rathcke (2009) 0.81 (0.66, 1.00) 13.00
Carole Ober (2008) 0.55 (0.39, 0.77) 11.51
Carole Ober (2008) 0.50 (0.29, 0.86) 8.95
Subtotal (I-squared = 73.2%, p = 0.005) 0.79 (0.58, 1.08) 52.39
African
Kamleshun Ramphul (2015) 1.01 (0.66, 1.55) 10.33
Subtotal (I-squared = .%, p = .) 1.01 (0.66, 1.55) 10.33
Asian
Ji-Ming LI (2015) 1.77 (1.24, 2.52) 11.26
Jin-lian Shao (2013) 1.53 (1.03, 2.26) 10.81
Ya-Wang Shao (2017) 4.62 (1.34, 15.86) 3.54
Guo Chen (2019) 0.92 (0.67, 1.28) 11.67
Sarla Naglot (2015) (Excluded) 0.00
Subtotal (I-squared = 74.3%, p = 0.009) 1.52 (0.97, 2.36) 37.28
Overall (I-squared = 79.4%, p = 0.000) 1.04 (0.79, 1.35) 100.00
NOTE: Weights are from random effects analysis
.063
1
15.9

c

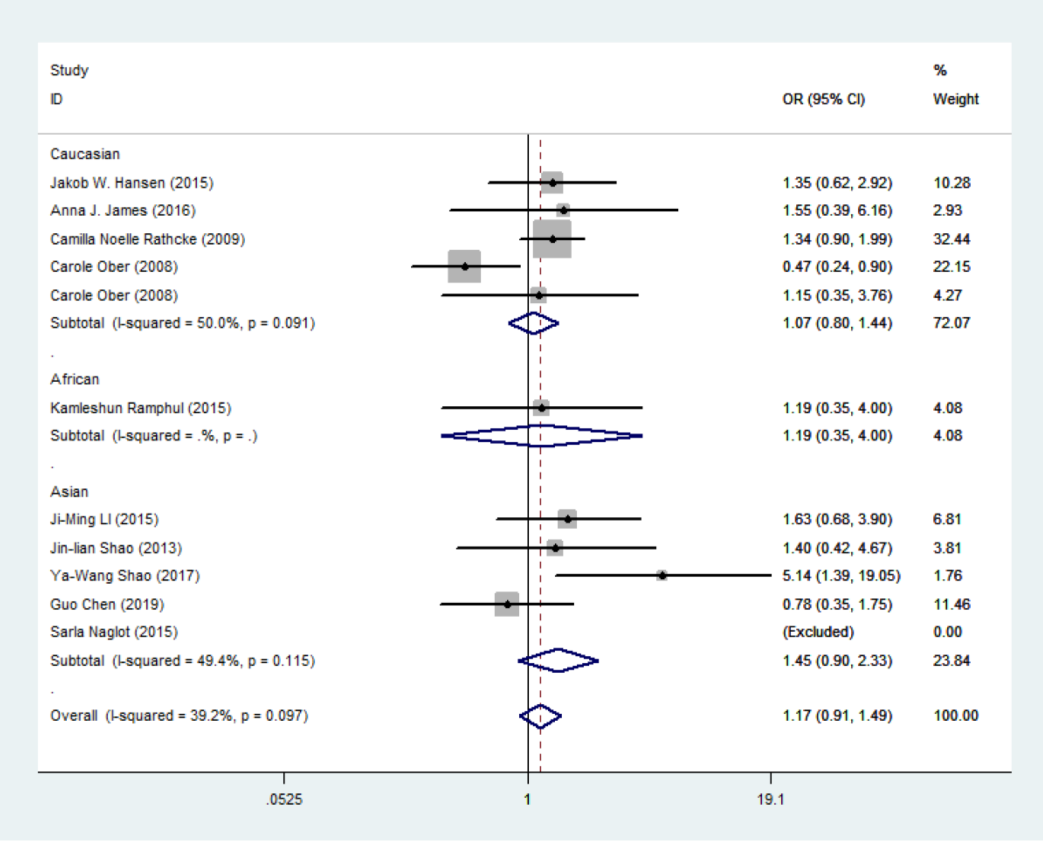
Study
ID
OR (95% CI)
%
Weight
Caucasian
Jakob W. Hansen (2015) 1.35 (0.62, 2.92) 10.28
Anna J. James (2016) 1.55 (0.39, 6.16) 2.93
Camilla Noelle Rathcke (2009) 1.34 (0.90, 1.99) 32.44
Carole Ober (2008) 0.47 (0.24, 0.90) 22.15
Carole Ober (2008) 1.15 (0.35, 3.76) 4.27
Subtotal (I-squared = 50.0%, p = 0.091) 1.07 (0.80, 1.44) 72.07
African
Kamleshun Ramphul (2015) 1.19 (0.35, 4.00) 4.08
Subtotal (I-squared = .%, p = .) 1.19 (0.35, 4.00) 4.08
Asian
Ji-Ming LI (2015) 1.63 (0.68, 3.90) 6.81
Jin-lian Shao (2013) 1.40 (0.42, 4.67) 3.81
Ya-Wang Shao (2017) 5.14 (1.39, 19.05) 1.76
Guo Chen (2019) 0.78 (0.35, 1.75) 11.46
Sarla Naglot (2015) (Excluded) 0.00
Subtotal (I-squared = 49.4%, p = 0.115) 1.45 (0.90, 2.33) 23.84
Overall (I-squared = 39.2%, p = 0.097) 1.17 (0.91, 1.49) 100.00
.0525
1
19.1

d

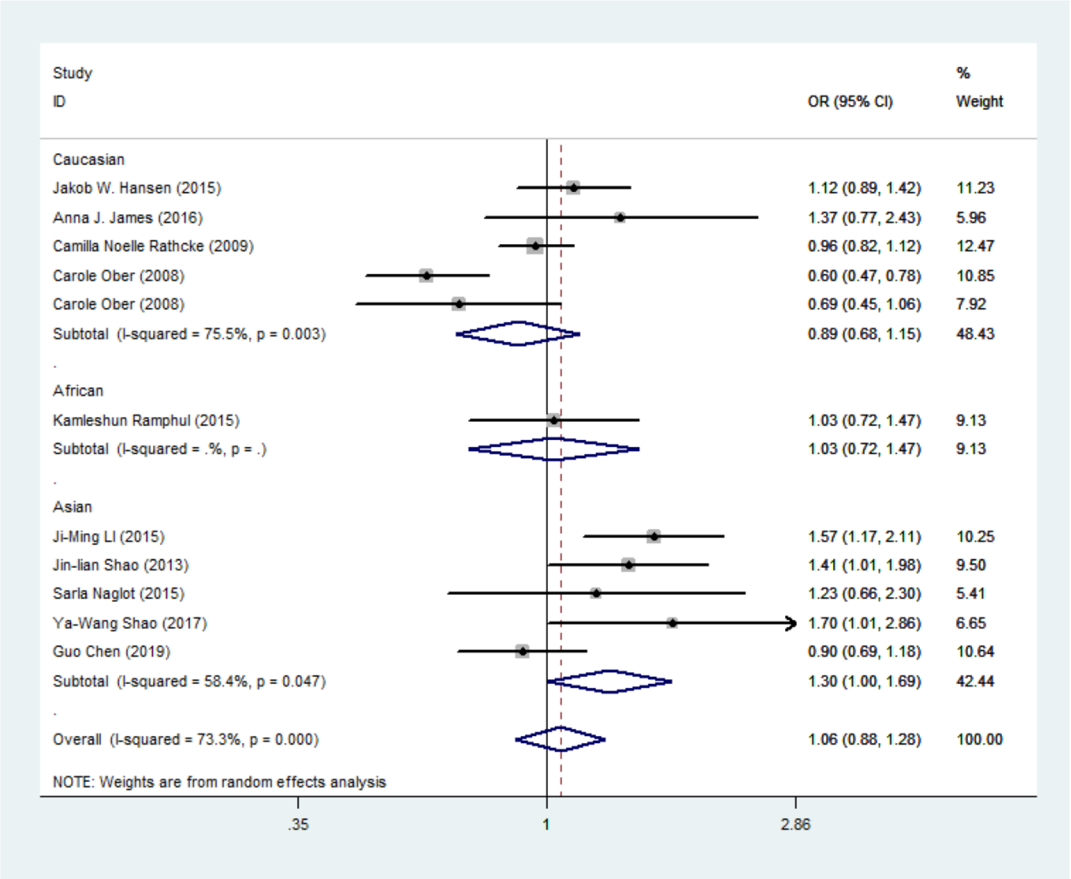

e

Figure S3 Forest plot of the association between *CHI3L1* rs4950928 and asthma by ethnicity stratification under different models. a, the recessive model; b, dominant model; c, codominant (GC vs. CC); d, codominant (GG vs. CC); e, allele model.

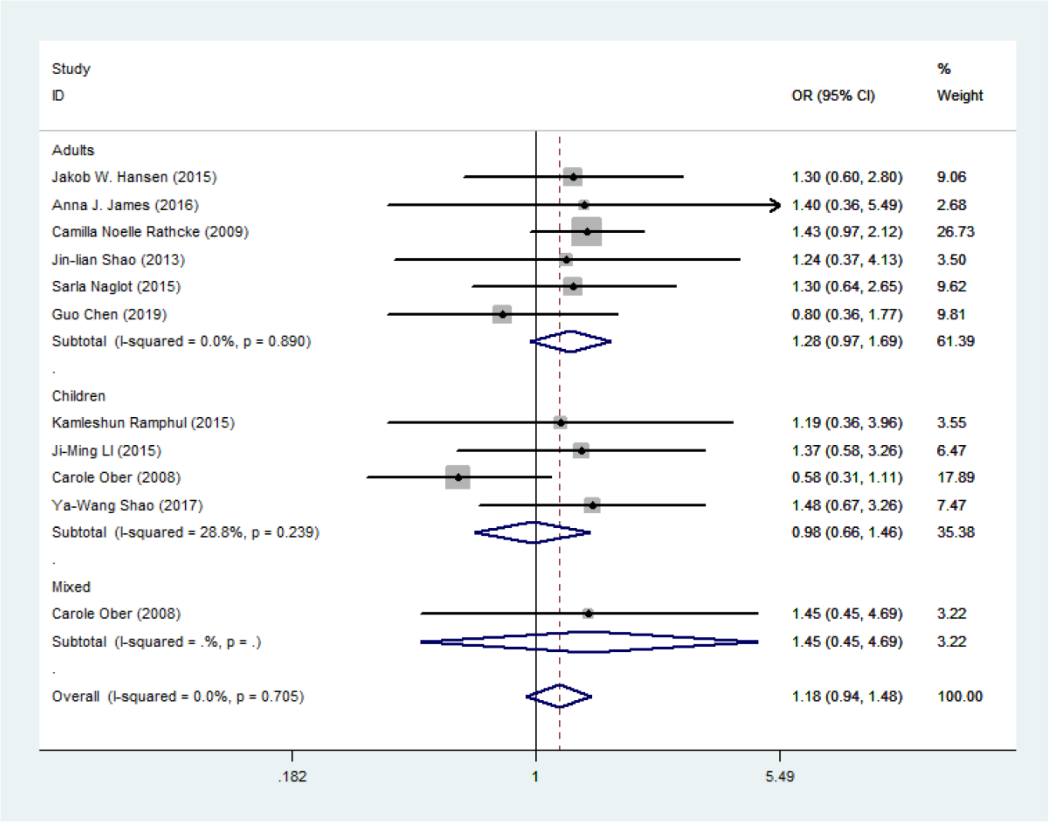

Study
ID
OR (95% CI)
% Weight
Adults
Jakob W. Hansen (2015) 1.30 (0.60, 2.80) 9.06
Anna J. James (2016) 1.40 (0.36, 5.49) 2.68
Camilla Noelle Rathcke (2009) 1.43 (0.97, 2.12) 26.73
Jin-lian Shao (2013) 1.24 (0.37, 4.13) 3.50
Sarla Naglot (2015) 1.30 (0.64, 2.65) 9.62
Guo Chen (2019) 0.80 (0.36, 1.77) 9.81
Subtotal (I-squared = 0.0%, p = 0.890) 1.28 (0.97, 1.69) 61.39
Children
Kamleshun Ramphul (2015) 1.19 (0.36, 3.96) 3.55
Ji-Ming LI (2015) 1.37 (0.58, 3.26) 6.47
Carole Ober (2008) 0.58 (0.31, 1.11) 17.89
Ya-Wang Shao (2017) 1.48 (0.67, 3.26) 7.47
Subtotal (I-squared = 28.8%, p = 0.239) 0.98 (0.66, 1.46) 35.38
Mixed
Carole Ober (2008) 1.45 (0.45, 4.69) 3.22
Subtotal (I-squared = .%, p = .) 1.45 (0.45, 4.69) 3.22
Overall (I-squared = 0.0%, p = 0.705) 1.18 (0.94, 1.48) 100.00
.182
1
5.49

a

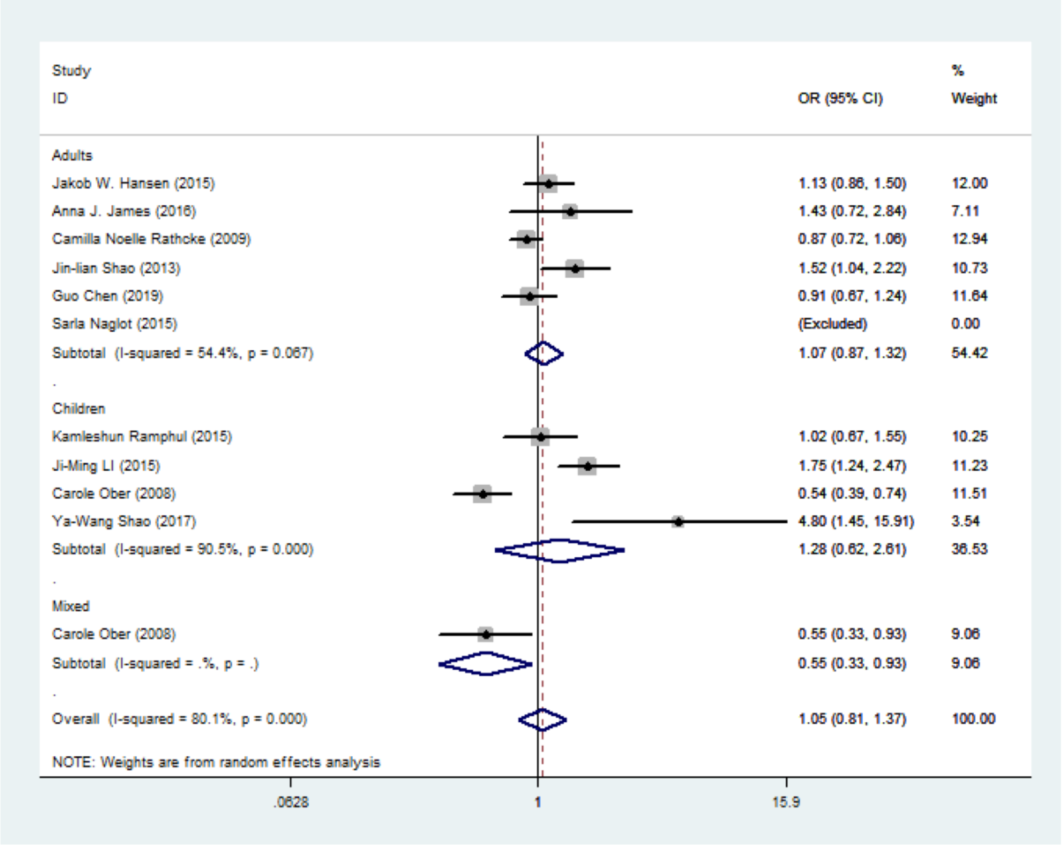

Study
ID
OR (95% CI)
% Weight
Adults
Jakob W. Hansen (2015) 1.13 (0.86, 1.50) 12.00
Anna J. James (2016) 1.43 (0.72, 2.84) 7.11
Camilla Noelle Rathcke (2009) 0.87 (0.72, 1.06) 12.94
Jin-lian Shao (2013) 1.52 (1.04, 2.22) 10.73
Guo Chen (2019) 0.91 (0.67, 1.24) 11.64
Sarla Naglot (2015) (Excluded) 0.00
Subtotal (I-squared = 54.4%, p = 0.067) 1.07 (0.87, 1.32) 54.42
Children
Kamleshun Ramphul (2015) 1.02 (0.67, 1.55) 10.25
Ji-Ming LI (2015) 1.75 (1.24, 2.47) 11.23
Carole Ober (2008) 0.54 (0.39, 0.74) 11.51
Ya-Wang Shao (2017) 4.80 (1.45, 15.91) 3.54
Subtotal (I-squared = 90.5%, p = 0.000) 1.28 (0.62, 2.61) 36.53
Mixed
Carole Ober (2008) 0.55 (0.33, 0.93) 9.06
Subtotal (I-squared = .%, p = .) 0.55 (0.33, 0.93) 9.06
Overall (I-squared = 80.1%, p = 0.000) 1.05 (0.81, 1.37) 100.00
NOTE: Weights are from random effects analysis
.0628
1
15.9

b

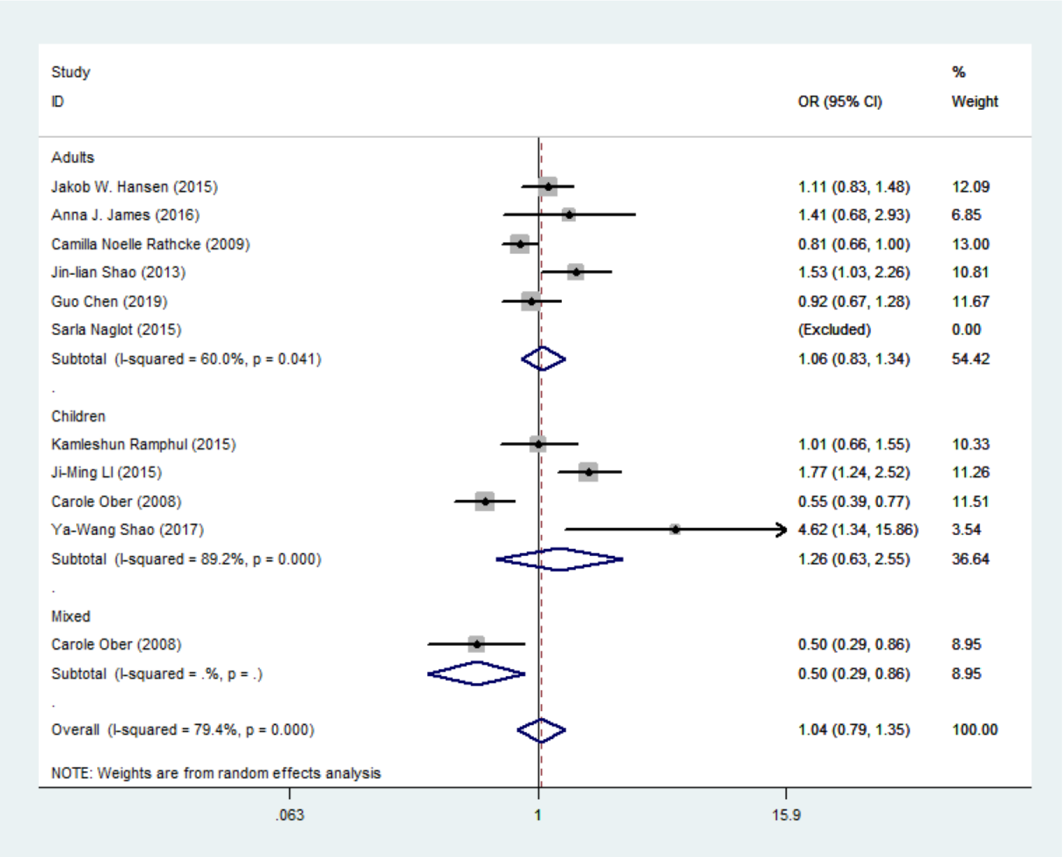
Study
ID
OR (95% CI)
%
Weight
Adults
Jakob W. Hansen (2015) 1.11 (0.83, 1.48) 12.09
Anna J. James (2016) 1.41 (0.68, 2.93) 6.85
Camilla Noelle Rathcke (2009) 0.81 (0.66, 1.00) 13.00
Jin-lian Shao (2013) 1.53 (1.03, 2.26) 10.81
Guo Chen (2019) 0.92 (0.67, 1.28) 11.67
Sarla Naglot (2015) (Excluded) 0.00
Subtotal (I-squared = 60.0%, p = 0.041) 1.06 (0.83, 1.34) 54.42
.
Children
Kamleshun Ramphul (2015) 1.01 (0.66, 1.55) 10.33
Ji-Ming LI (2015) 1.77 (1.24, 2.52) 11.26
Carole Ober (2008) 0.55 (0.39, 0.77) 11.51
Ya-Wang Shao (2017) 4.62 (1.34, 15.86) 3.54
Subtotal (I-squared = 89.2%, p = 0.000) 1.26 (0.63, 2.55) 36.64
.
Mixed
Carole Ober (2008) 0.50 (0.29, 0.86) 8.95
Subtotal (I-squared = .%, p = .) 0.50 (0.29, 0.86) 8.95
.
Overall (I-squared = 79.4%, p = 0.000) 1.04 (0.79, 1.35) 100.00
NOTE: Weights are from random effects analysis
.063
1
15.9

c

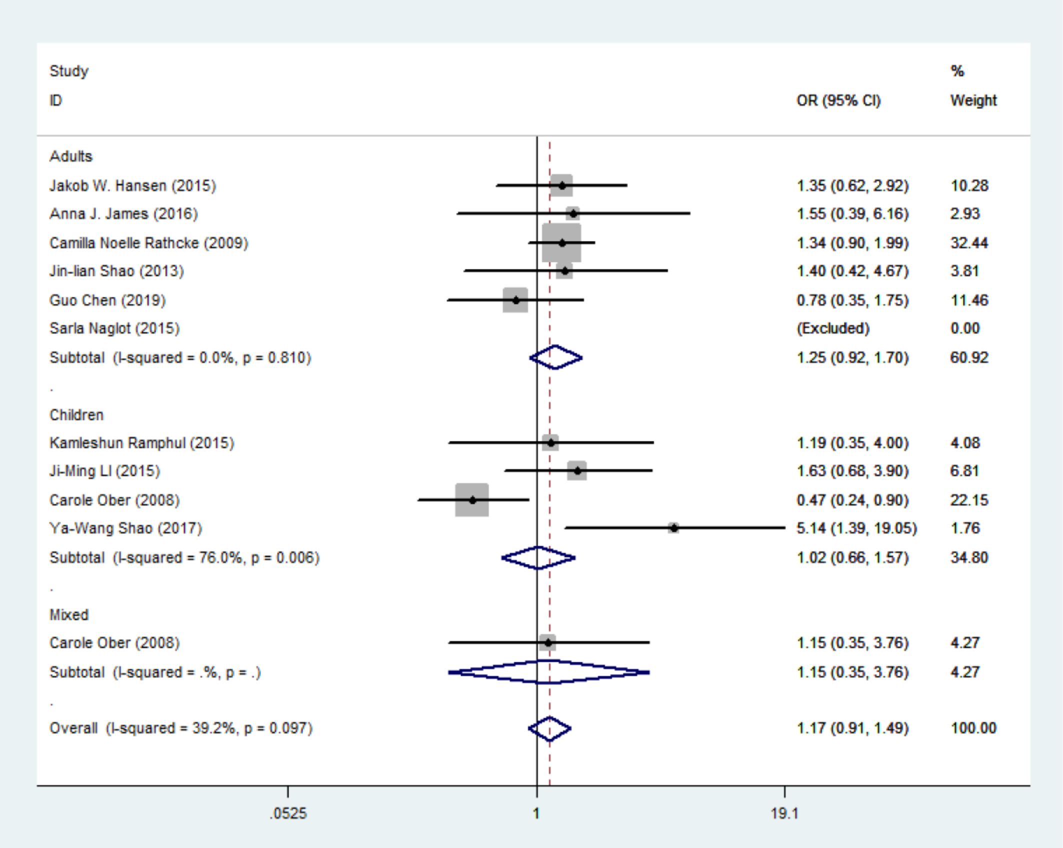
Study
ID
OR (95% CI)
%
Weight
Adults
Jakob W. Hansen (2015) 1.35 (0.62, 2.92) 10.28
Anna J. James (2016) 1.55 (0.39, 6.16) 2.93
Camilla Noelle Rathcke (2009) 1.34 (0.90, 1.99) 32.44
Jin-lian Shao (2013) 1.40 (0.42, 4.67) 3.81
Guo Chen (2019) 0.78 (0.35, 1.75) 11.46
Sarla Naglot (2015) (Excluded) 0.00
Subtotal (I-squared = 0.0%, p = 0.810) 1.25 (0.92, 1.70) 60.92
.
Children
Kamleshun Ramphul (2015) 1.19 (0.35, 4.00) 4.08
Ji-Ming LI (2015) 1.63 (0.68, 3.90) 6.81
Carole Ober (2008) 0.47 (0.24, 0.90) 22.15
Ya-Wang Shao (2017) 5.14 (1.39, 19.05) 1.76
Subtotal (I-squared = 76.0%, p = 0.006) 1.02 (0.66, 1.57) 34.80
.
Mixed
Carole Ober (2008) 1.15 (0.35, 3.76) 4.27
Subtotal (I-squared = .%, p = .) 1.15 (0.35, 3.76) 4.27
.
Overall (I-squared = 39.2%, p = 0.097) 1.17 (0.91, 1.49) 100.00
.0525
1
19.1

d

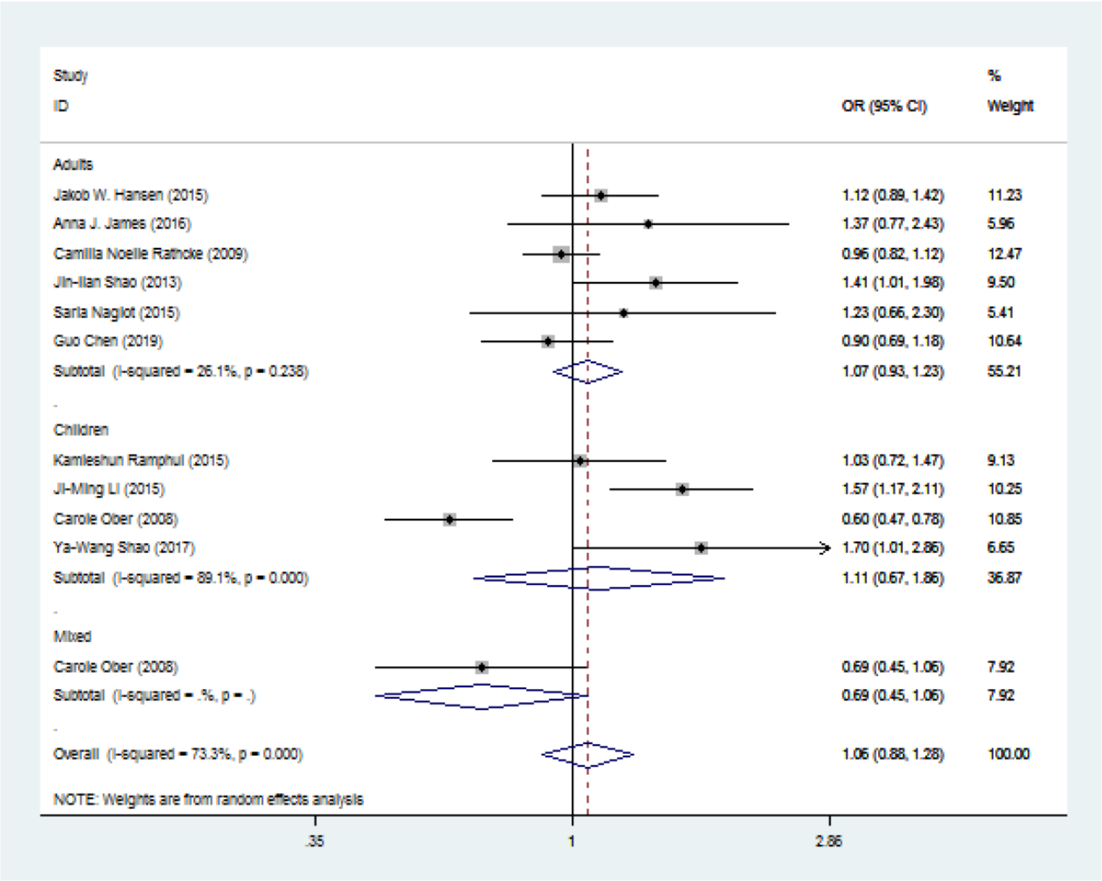

e

Figure S4 Forest plot of the association between *CHI3L1* rs4950928 and asthma by age stratification under different models. a, the recessive model; b, dominant model; c, codominant (GC vs. CC); d, codominant (GG vs. CC); e, allele model.

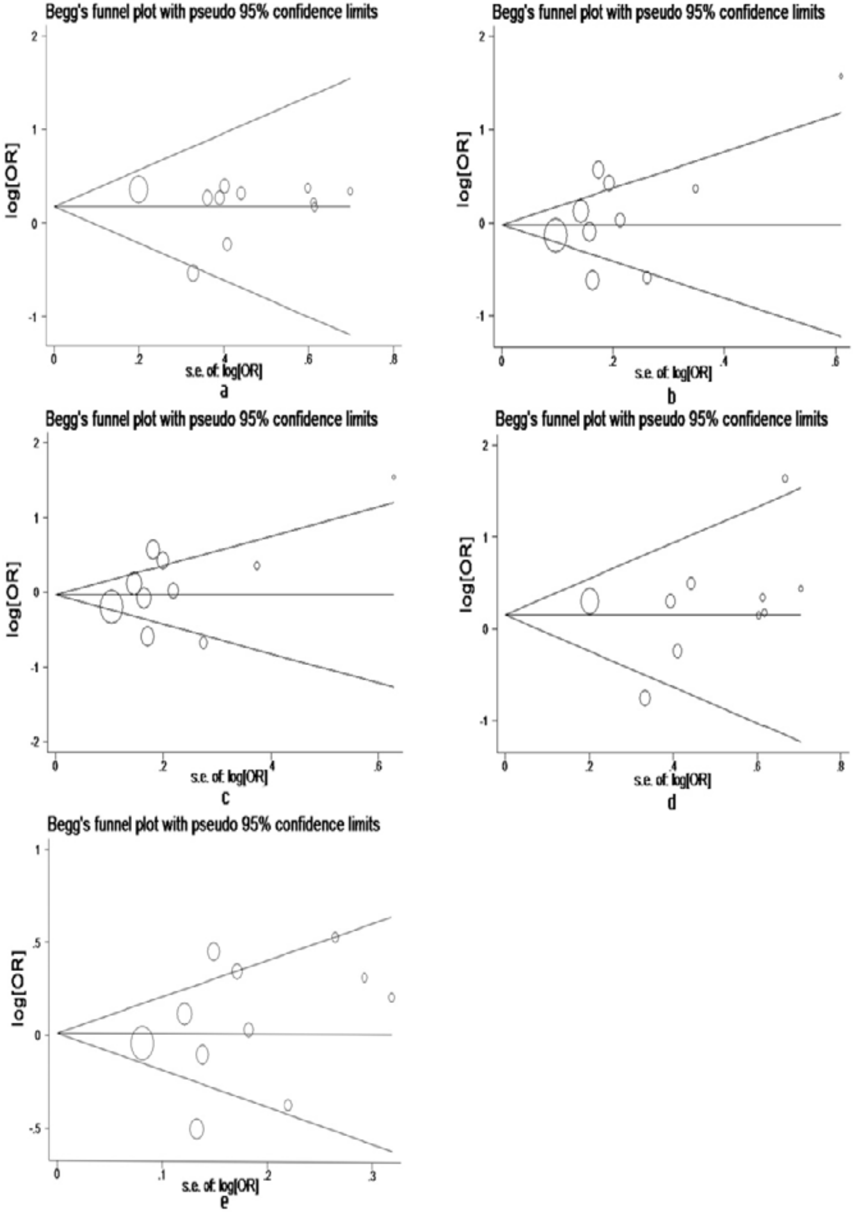

Figure S5 Begg’s funnel plots of publication bias for all studies
a, recessive model; b, dominant model; c, codominant (GC vs. CC); d, codominant (GG vs. CC); e, allele model.
